# Double Strand Breaks and Cell-Cycle Arrest Induced by the Cyanobacterial Toxin Cylindrospermopsin in HepG2 Cells

**DOI:** 10.3390/md11083077

**Published:** 2013-08-21

**Authors:** Alja Štraser, Metka Filipič, Matjaž Novak, Bojana Žegura

**Affiliations:** Department of Genetic Toxicology and Cancer Biology, National Institute of Biology, Večna pot 111, Ljubljana 1000, Slovenia; E-Mails: alja.straser@nib.si (A.Š.); metka.filipic@nib.si (M.F.); matjaz.novak@nib.si (M.N.)

**Keywords:** cylindrospermopsin, cell-cycle, cell-proliferation, double-strand breaks, HepG2 cells

## Abstract

The newly emerging cyanobacterial cytotoxin cylindrospermopsin (CYN) is increasingly found in surface freshwaters, worldwide. It poses a potential threat to humans after chronic exposure as it was shown to be genotoxic in a range of test systems and is potentially carcinogenic. However, the mechanisms of CYN toxicity and genotoxicity are not well understood. In the present study CYN induced formation of DNA double strand breaks (DSBs), after prolonged exposure (72 h), in human hepatoma cells, HepG2. CYN (0.1–0.5 µg/mL, 24–96 h) induced morphological changes and reduced cell viability in a dose and time dependent manner. No significant increase in lactate dehydrogenase (LDH) leakage could be observed after CYN exposure, indicating that the reduction in cell number was due to decreased cell proliferation and not due to cytotoxicity. This was confirmed by imunocytochemical analysis of the cell-proliferation marker Ki67. Analysis of the cell-cycle using flow-cytometry showed that CYN has an impact on the cell cycle, indicating G0/G1 arrest after 24 h and S-phase arrest after longer exposure (72 and 96 h). Our results provide new evidence that CYN is a direct acting genotoxin, causing DSBs, and these facts need to be considered in the human health risk assessment.

## 1. Introduction

The cyanobacterial toxin cylindrospermopsin (CYN) is synthesized by a number of freshwater cyanobacterial species (for review see: [1]) and is increasingly being recognized as a potential threat to drinking water safety, worldwide. The toxin is a stable 415 Da tricyclic polyketide-derived alkaloid, containing a guanido group linked at C7 to hydroxymethyl uracil through a hydroxyl bridge [2]. CYN was first identified as the probable cause of a severe case of human poisoning in Australia in 1979 [3], and was since then found to be implicated in several cases of human intoxications and animal mortality (for review see: [4]). It was first thought to be primarily associated with liver damage, but is now considered a cytotoxic and genotoxic toxin, due to its effects in other organs such as the kidneys, lungs, thymus, spleen, adrenal glands, intestinal tract, the immune system and the heart [5], and on DNA (for review see: [4]), respectively.

The toxin is a potent protein synthesis inhibitor [6], and contains several potential sites for reactivity that may form protein and DNA adducts. There is evidence for its genotoxic activity *in vitro* [7,8,9,10,11] and *in vivo* [12,13], and even carcinogenic potential of CYN has been indicated by preliminary results [14]. The majority of the studies show that CYN is a pro-genotoxin that needs to be activated by enzymes from the cytochome P450 (CYP450) family [7,10,11]. However, despite of its apparent hazard, the mechanisms involved in CYN genotoxic and especially carcinogenic activity are poorly understood. Therefore, the U.S. Environmental Protection Agency (EPA) classified CYN on the list of compounds with highest priority for hazard characterization [15]. The World Health Organisation (WHO) included CYN in the revision of the WHO “Guidelines for Drinking-water Quality, chemical hazards in drinking-water”, but there is still insufficient information for the classification of CYN as a carcinogen by the International Agency for Research on Cancer (IARC).

Its protein synthesis inhibition ability and its genotoxic activity suggest that CYN has an impact on cell-proliferation and cell-cycle progression. The first response upon DNA damage is cell-cycle checkpoint activation, delaying cell-cycle progression and allowing cells to repair defects, thus preventing their transmission to the daughter cells [16]. In addition, the protein synthesis inhibition correlates with decrease in cellular proliferation and influences the onset and completion of mitosis [17,18]. Nevertheless, limited data has been published regarding this topic in mammalian test systems. Therefore, the aim of this study was to investigate the influence of CYN on cell-proliferation and cell-cycle progression in the metabolically active human hepatoma cell line, HepG2.

## 2. Results and Discussion

It is generally accepted that CYN is genotoxic as it induces DNA damage in several *in vitro* [7,8,9,10,11,19] and *in vivo* test systems [12,13]. In the present study the formation of DNA double strand breaks (DSBs) by CYN was shown for the first time. In addition, the influence of genotoxic CYN concentrations on the cell-cycle and cell-proliferation in HepG2 cells was shown.

### 2.1. Viability of HepG2 Cells after CYN Exposure

**Figure 1:**
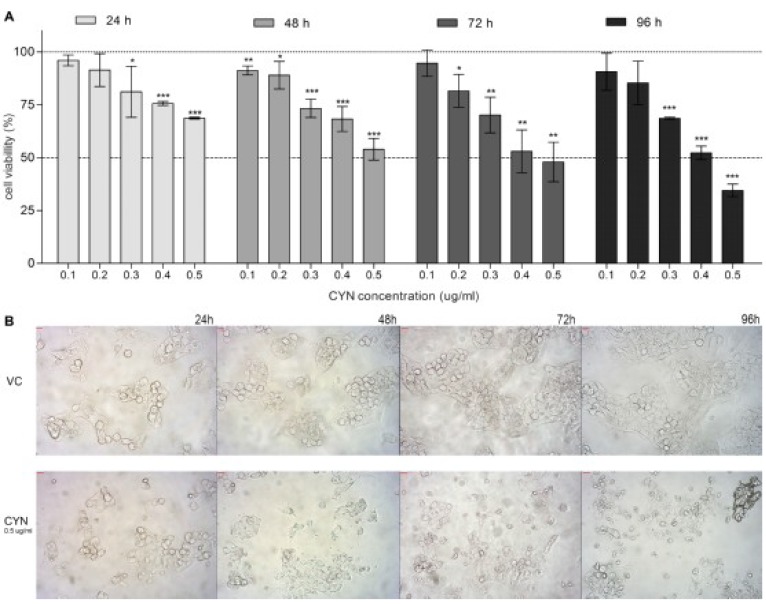
Cylindrospermopsin (CYN) exposure reduces cell viability. HepG2 cells were incubated for 24, 48, 72 and 96 h with CYN (0.1, 0.2, 0.3, 0.4 and 0.5 µg/mL) and cell viability was assessed by the MTT assay. In each experiment, a vehicle control (VC, 0.05% methanol) was included. (**A**) Relative viability of cells is shown; the vehicle control was regarded as 100%. Significant difference between CYN-treated cells and the vehicle control is indicated by * *p* < 0.05, ** *p* < 0.01 and *** *p* < 0.001. (**B**) Micrographs of cells from the vehicle control group (VC) and cells exposed to 0.5 µg/mL CYN under the microscope (magnified 200-times) at every experimental point. Independent experiments were performed in multiple replicates and were repeated at least three times.

CYN significantly affected cell viability in a dose and time dependent manner (Figure 1A). After 24 h of exposure, significant decrease in cell viability was detected at the concentration 0.3 µg/mL and above, however the cell survival at the highest tested concentration was still more than 70%. After longer exposure (96 h), CYN reduced cell viability for about 50% to up to 65% at the concentrations 0.4 and 0.5 µg/mL, respectively. The toxin (0.5 µg/mL) induced morphological changes that were observed under the light microscope (Figure 1B) especially after longer exposure (from 48 h onwards). 

**Figure 2:**
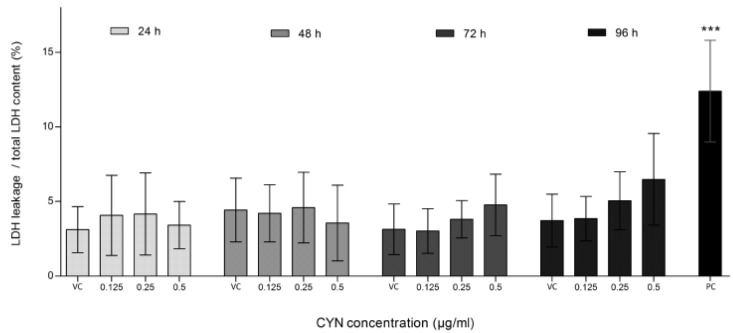
The influence of CYN on cell death. Cell death was assessed by lactate dehydrogenase (LDH) leakage. LDH content in the medium was determined spectrophotometrically using the Cytotox-ONE Homogenous Membrane Integrity Assay (Promega). LDH leakage after exposure to CYN (0.125, 0.25 and 0.5 µg/mL) for 24, 48, 72 and 96 h, and total LDH content at each experimental point was measured. In each experiment a vehicle control (VC, 0.05% methanol) and a positive control (PC, 0.1 μM staurosporine) were included. The amount of the fluorescent product is proportional to the number of death cells. The percentage of LDH leakage from total LDH content is shown. Significant difference between CYN-treated cells and the vehicle control (VC) is indicated by * *p* < 0.05, ** *p* < 0.01 and *** *p* < 0.001. Independent experiments were performed in multiple replicates and were repeated at least three times.

There was no significant increase in lactate dehydrogenase (LDH) leakage in cells exposed to CYN at any of the tested time-points and concentrations, moreover a decrease in LDH leakage was observed. However, total LDH content also decreased and was significantly different after 24 h (0.5 µg/mL), 48 h (0.25 and 0.5 µg/mL), 72 h (0.125 and 0.5 µg/mL) and 96 h (0.5 µg/mL) of exposure, again indicating decreased cell number. Therefore, when calculating the ratio between LDH leakage and total LDH content in the sample (LDH leakage/total), the ratio remained at the control level (Figure 2). These findings show that the reduced cell number after CYN exposure is not due to cytotoxicity but rather due to decreased cell proliferation. This correlates with our previous study on HepG2 cells, showing no apoptosis induction after CYN exposure [20]. Our results are also supported by the findings from Fessard and Bernard [21] and Lankoff *et al*. [22], who reported decrease in the number of mitotic figures and decrease in the mitotic index and proliferation in CHO-K1 cells exposed to CYN, respectively. In addition, in lymphoblastoid WIL2-NS cells exposed to CYN, a dose-dependent inhibition of cell division was observed [9], while in HepG2 cells [10] and in human peripheral blood lymphocytes (HPBLs) [11] CYN significantly decreased the nuclear division index.

### 2.2. Formation of DNA Double Strand Beaks (DSBs) by CYN

DSBs are the most detrimental form of DNA damage as they can lead to chromosomal breakage and rearrangement [23]. CYN is expected to form DSBs as it was shown to be a clastogenic and aneugenic agent causing micronuclei *in vivo* and *in vitro* [7,8,10,11,13]. Induction of DSBs initiates fine-tuned networks that lead to repair by homologous recombination (HRR) or non-homologous end joining (NHEJ), checkpoint activation and cell-cycle arrest, apoptosis mostly via P53, activation of MAPKs, and the transcription factors AP-1 and NF-κB [24,25]. Involvement of P53, AP-1 and NF-κB signaling in the cellular response to CYN was indicated in our previous study on the transcriptional response of HepG2 cells to CYN exposure [19]. CYN was also shown to deregulate several genes involved in DSB repair in HepG2 cells [19].

**Figure 3:**
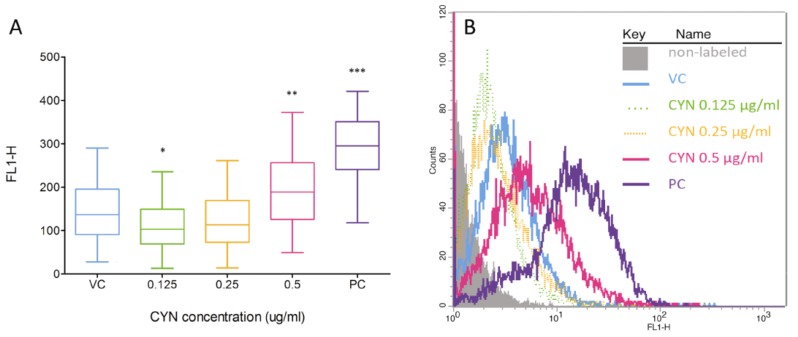
Induction of double strand breaks (DSB) by CYN. HepG2 cells were incubated for 72 h with CYN (0.125, 0.25 and 0.5 µg/mL) and the presence of DSB was analyzed by flow cytometry, indirectly through the detection of γH2A.X foci. In each experiment a vehicle control (VC, 0.05% methanol) and a positive control (PC, 1 µg/mL etoposide, 24 h) were included. (**A**) Distribution of the fluorescent signals of individual cells in the samples is shown. Data are presented as quantile box plots. The edges of the box represent the 25th and 75th percentiles, the median is a solid line through the box, and the bars represent 95% confidence intervals. In each sample, 10^4^ events were recorded and experiments were repeated three times. Significant difference between CYN-treated cells and the vehicle control (0) is indicated by * *p* < 0.05, ** *p* < 0.01 and *** *p* < 0.001. (**B**) Representative histograms are shown.

DSBs induction is rapidly followed by phosphorylation of the histone, H2AX [26], which is a component of the histone octomer in nucleosomes [27]. The phosphorylated H2AX histones (γH2AX) accumulate at sites of the DSBs, forming foci that correlate to DSBs within a 1:1 ratio [27,28], and can therefore be used as a biomarker for DSBs and DNA damage. For the first time the presence of DSBs induced by CYN was analyzed by flow cytometry, by measuring the fluorescent signals of individual cells, indirectly through the detection of γH2AX foci. After 24 h of exposure, there was no increase in DSB formation (data not shown), while after prolonged exposure (72 h) CYN significantly induced DSBs at 0.5 µg/mL (Figure 3). At 0.125 µg/mL CYN significantly decreased the γH2AX signal compared to the control, the same was observed after 24 h of exposure to 0.125 and 0.25 µg/mL CYN (data not shown). This could be due to DSB repair processes or the protein synthesis inhibition by CYN; however, it needs to be further elucidated.

### 2.3. Influence of CYN on Cell Proliferation

**Figure 4:**
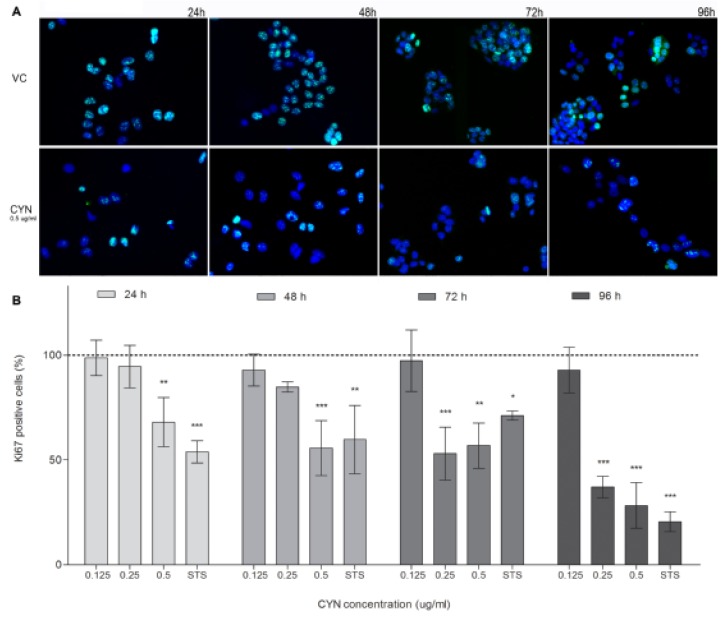
CYN influence on Ki67 expression. HepG2 cells were incubated for 24, 48, 72 and 96 h with CYN (0.125, 0.25 and 0.5 µg/mL) and imunocytochemical staining of Ki67 was performed. In each experiment a vehicle control (VC, 0.05% methanol) and a positive control (PC, 0.1 μM staurosporine) were included. Five hundreds (500) nuclei were counted under the fluorescent microscope and percentage of Ki67 positive cells was assessed. (**A**) Representative fluorescent micrographs of the vehicle control (VC) and CYN (0.5 µg/mL) exposed cells are shown (magnified 460-times). (**B**) Significant difference between CYN-treated cells and the vehicle control (VC) is indicated by * *p* < 0.05, ** *p* < 0.01 and *** *p* < 0.001. Independent experiments were performed three times.

In addition to the measurement of cell viability and total LDH content, the influence of CYN on cell proliferation inhibition was analyzed by the detection of cells positive for the proliferation marker Ki67. CYN decreased the percentage of Ki67 positive cells at all exposure times (Figure 4). After 24 and 48 h of exposure there was a statistically significant decrease at the highest tested concentration (0.5 µg/mL), while after longer exposure (72 and 96 h), the decrease in Ki67 positive cells was statistically significant already at 0.25 µg/mL. At the concentration 0.125 µg/mL CYN had no effect on the expression of Ki67 at any time point. The expression of the human Ki67 protein is strictly associated with cell proliferation as the protein is present during all active phases of the cell cycle (G1, S, G2, and M), and absent from resting cells (G0) [29]. Decrease in Ki67 positive cells could also be a direct consequence of CYN induced protein synthesis inhibition as reduction in Ki67 staining intensity after inhibition of protein synthesis was reported before [30]. However, as the Ki76 expression decrease correlated with the decrease in viable cells, and number of cells seen under the microscope as well as the total LDH content, it probably reflects reduced cell proliferation. Nevertheless, contribution of the protein synthesis inhibition to the decreased expression of Ki67 cannot be excluded.

### 2.4. Influence on the Cell-Cycle Progression

**Figure 5:**
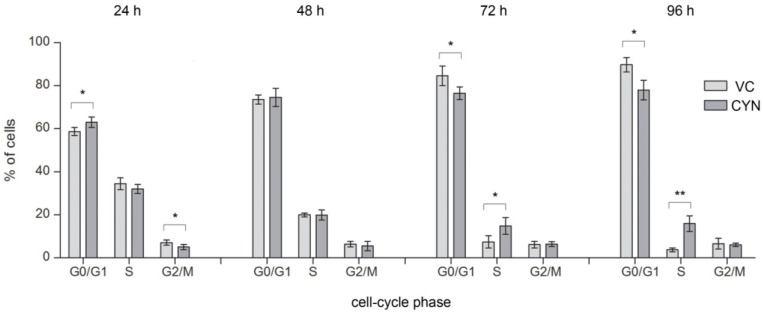
CYN influences cell-cycle phase distribution of the exposed cell population. HepG2 cells were incubated for 24, 48, 72 and 96 h with CYN (0.5 µg/mL) and cell-cycle analysis was performed by flow-cytometry using propidium iodide (PI) staining. In each experiment a vehicle control (VC, 0.05% methanol) was included. The percentage of cells in G0/G1, S, and G2/M phases of the cell cycle was determined from FL2-A histograms using ModFit LT™ (version 3.3). Charts represent differences between cell distribution through the cell-cyle phases in CYN-treated cells and the vehicle control (VC), after 24, 48, 72 and 96 h. Significant difference is indicated by * *p* < 0.05 and ** *p* < 0.01. In each sample, 10^4^ events were recorded and experiments were repeated three times.

For the first time it was shown that CYN (0.5 µg/mL) exposure affected the distribution of the cells through the cell-cycle (Figure 5). After 24 h CYN (0.5 µg/mL) significantly increased the amount of cells in G0/G1 phase and decreased the percentage of cells in G2/M phase, compared to the control population, indicating prevention of cells from entering S phase or even committing to cell division in the first place. It is well known that DNA damage can induce G1 phase arrest through P53 and CDKN1A (for reviews see [31,32]). Induction of G1 phase arrest after CYN exposure (12 and 24 h) was also indicated by gene expression studies in HepG2 cells [19] and the involvement of P53 signaling and CDKN1A in the cellular response to CYN, supporting this findings, was already indicated by the up-regulation of CDKN1A and other P53 downstream regulated genes in HepG2 cells [10,19,33], in HPBLs [11] and human dermal fibroblasts (HDFs) [33]. Cell cycle arrest was also observed after 48 h although to a lesser extent and the changes were not statistically significant. Lower CYN concentrations (0.125 and 0.25 µg/mL) did not induce statistically significant changes in the cell-cycle distribution of cells, at any of the exposure times (data not shown). The positive control (STS 0.5 µM) significantly decreased the percentage of cells in the G0/G1 and increased the percentage of cells in the G2/M phase (G0/G1 = 35.24 ± 5.87; S = 27.88 ± 2.28; G2/M = 36.88 ± 3.59).

After longer exposure, the percentage of cells in S phase started to increase in a dose dependent manner (in Figure 5 only the results at concentration 0.5 µg/mL are presented) and was significant after 72 and 96 h exposure to 0.5 µg/mL CYN (Figure 5). The increase of cells in S phase was accompanied by decrease in G0/G1 phase cells, indicating cell-cycle arrest by CYN in the S phase. There are three checkpoints in S phase: the replication checkpoint and the S/M checkpoint, which both respond to DNA replication errors, and the replication-independent intra-S-phase checkpoint that is induced in response to DNA double strand breaks (DSBs) [16]. It is assumed that CYN can bind to DNA as it contains sulfate, guanidine and uracil groups. Covalent binding of CYN or its metabolites to DNA in mice [12] and DNA strand breakage [13] have already been reported. In the present study the formation of DSBs after prolonged exposure to CYN was shown, which correlated with the time point of the S phase arrest. These observations are also supported by the results of our previous study where CYN deregulated several genes involved in nucleotide excision repair (NER), which repairs DNA adducts, and DSB repair genes in HepG2 cells [19]. Replication errors after CYN exposure were also indicated by several studies, that in addition to increased frequencies of micronuclei (MNi) [7,9,10,11] reported appearance of other irregular DNA structures such as nucleoplasmic bridges (NPBs) and nuclear buds (NBUDs) [10,11], arising from extrusions of either amplified DNA [34] or chromatin, whose replication has failed during S-phase [35] and DNA miss-repair, chromosome rearrangements or telomere end-fusions [36], respectively.

## 3. Experimental Section

### 3.1. Chemicals

Cylindrospermopsin (CYN) was from Enzo Life Sciences GmbH, Lausen, Switzerland. A 0.5 mg/mL stock solution of CYN was prepared in 50% methanol. William’s medium E, methanol, 3-(4,5-dimethylthiazol-2-yl)-2,5-diphenyltetrazolium bromide (MTT), dimethylsulphoxide (DMSO) and staurosporine (STS) were obtained from Sigma, St. Louis, MO, USA. Penicillin/streptomycin, foetal bovine serum (FBS), l-glutamine and phosphate buffered saline (PBS) were from PAA Laboratories, Dartmouth, NH, USA. Trypsin was from BD-Difco, Le Pont-De-Claix Cedex, France. Mouse monoclonal IgG1, Anti-phospho-Histone H2A.X (Ser139), FITC conjugate, were from Milipore, Billerica, MA, USA. Alexa Fluor^®^ 488 Goat Anti-Rabbit antibodies were from Invitrogen™, Life Technologies, Carlsbad, CA, USA. Rabbit anti-Ki67 polyclonal antibodies were from Abcam^®^, Cambridge, UK. All other chemical reagents were of the purest grade available and all solutions were made using Milli-Q water.

### 3.2. Cell Culture

HepG2 cells were a gift from Dr. Firouz Darroudi (Leiden University Medical Centre, Department of Toxicogenetics, Leiden, The Netherlands). The cells were grown at 37 °C and 5% CO2 in William’s medium E containing 15% foetal bovine serum, 2 mM l-glutamine and 100 U/mL penicillin/streptomycin.

### 3.3. Cell Viability—MTT Assay

Cell viability after exposure to CYN was determined with 3-(4,5-dimethylthiazol-2-yl)-2,5 diphenyltetrazolium bromide (MTT) according to Mosmann [37] with minor modifications [38]. The HepG2 cells were seeded onto 96-well microplates (Nunc, Naperville, IL, USA) at a density of 3000 cells/well and incubated for 4 h at 37 °C in 5% CO2 to attach. Fresh medium containing CYN was added to the wells to gain final concentrations of 0.1, 0.2, 0.3, 0.4 and 0.5 μg/mL. In each experiment, a vehicle control (0.05% methanol) was included. Measurements were taken after 24, 48, 72 and 96 h after the exposure to CYN. Images of control and exposed cells were taken under the light microscope (Nikon, Diaphot, Tokyo, Japan) at each experimental point. Independent experiments were performed in 5 replicates and were repeated 3-times.

### 3.4. Lactate Dehydrogenase (LDH) Leakage

Lactate Dehydrogenase (LDH) Leakage was determined using CytoTox-ONE™ Homogeneous Membrane Integrity Assay. Cells were seeded on black 384 well plates (Corning Costar Corporation, Corning, NY, USA) at the density of 3000 cells/well in four replicates. After incubation at 37 °C in 5% CO2 for 24 h, the growth medium was replaced with fresh medium containing 0.125, 0.25 and 0.5 μg/mL CYN and cells were exposed to CYN for 24, 48, 72 and 96 h. In each experiment, a vehicle control (0.05% methanol) and a positive control (0.1 μM STS) were included. After the exposure, the assay was performed according to the manufacturers’ protocol with minor modifications. In brief, cells were left to cool to room temperature for 20 min and 25 µL of the CytotoxOne reagent was added to each well. The cells were shaken for 30 s and incubated for 10 min at room temperature. After that, 12.5 µL stop solution was added to each well and after shaking for 10 s the fluorescence was measured at 560 nm excitation and 590 nm emission, gain 55, using a microplate reading spectrofluorimeter (SynergyMx, BioTek, Winooski, VT, USA). Differences in cell number and growth after the exposure to CYN were assessed by parallel performance of the maximum LDH release control, determining total LDH content after total lysis of the cells, as described by the manufacturer. The maximum LDH release control was performed in the same way as the LDH leakage assay, with the sole difference that 0.5 µL of lysis solution was added in each well before the CytotoxOne reagent addition. The fluorescence was measured at 560 nm excitation and 590 nm emission, gain 55, using a microplate reading spectrofluorimeter (SynergyMx, BioTek, Winooski, VT, USA). Independent experiments were performed in 5 replicates and were repeated twice.

### 3.5. DSB Detection—H2AX Foci Analysis

The cells were seeded on T25 flasks at the density of 0.8 × 10^6^ per plate, left to attach overnight and exposed to CYN (0.125, 0.25 and 0.5 µg/mL) for 24 (short time exposure) and 72 h (long time exposure). In each experiment a vehicle control (0.05% methanol) and a positive control (1 µg/mL etoposide, 24 h) were included. At the end of the exposure floating and adherent cells were collected by trypsinization. For the fixation the cells were centrifuged at 800 rpm, 4 °C for 5 min, washed twice with ice cold PBS, resuspended in 0.5 mL cold PBS and ethanol (1.5 mL) was added drop wise into the cell pellet, while vortexing. The cells were fixed at 4 °C overnight and stored at −20 °C till analysis. Fixed cells were centrifuged at 1200 rpm for 10 min, washed twice with ice cold 1× PBS, resuspended in 0.5 mL 1× PBS containing 2000-fold diluted anti-γH2AX antibodies, mixed and incubated at 4 °C for 30 min in the dark. Labeled cells were then washed twice and resuspended in 0.3 mL of 1× PBS. Flow cytometric analysis was carried out on a FACSCalibur flow cytometer (BD Biosciences PharmingenTM, San Diego, CA, USA). FITC intensity, corresponding to DSBs, was detected in the FL1-H channel. In each sample, 10^4^ events were recorded. Independent experiments were repeated 3-times. For further analysis the raw data (FITC intensities of each cell, FL1-H intensity), obtained from the CellQuest Pro software (BD Biosciences), was converted from the .fcs to the .csv format, using the program FCSExtract, which is available on the website [39]. 

### 3.6. Cell-Proliferation—Immunocytochemical Staining of the Proliferation Marker Ki67

Cell-proliferation after CYN exposure was analyzed as described by Hreljac *et al*. [40], with minor modifications. Cells were seeded onto 24 well plates (Corning Costar Corporation, Corning, NY, USA), containing poly-l-lysine slips, at the density of 30,000 cells/well, left to attach overnight and exposed to 0.125, 0.25 and 0.5 μg/mL CYN for 24, 48, 72 and 96 h. In each experiment, a vehicle control (0.05% methanol) and a positive control (0.1 μM STS for 24 h) were included. The cells were fixed at room temperature (RT) in 100% methanol for 10 min, permeabilized with 0.1% Triton-X for 10 min, and blocked with 4% BSA for 15 min. Polyclonal anti-Ki67 rabbit antibodies, diluted 1:500 in PBS, were added to the cells and incubated for 1 h. Secondary anti-rabbit Alexa-Fluor 488 antibodies, diluted 1:500, were added and incubated for 90 min, all at RT. Hoechst (diluted 1:1000) was added for 5 min to stain the nuclei. Between the steps the slides were washed 5-times in PBS. After the staining anti-fade reagent was added on the object-slides and the slips were mounted on them and sealed. Slides were kept at −20 °C until scoring. Cells were scored under a fluorescence microscope (Nicon Eclipse Ti); pictures were obtained with FLoid^®^ Cell Imaging Station (Life technologies). Ki67 positive nuclei were granularly stained with intense green fluorescence. The percentage of proliferating cells was calculated as the ratio between Ki67 positive nuclei and total nuclei (stained with Hoechst). Five hundreds (500) cells were scored for each experimental point. Independent experiments were repeated 3-times.

### 3.7. Cell-Cycle Analysis by Flow Cytometry

HepG2 cells were seeded at a density of 400,000 cells/well into 6-well plates (Corning Costar Corporation, Corning, NY, USA). After incubation at 37 °C in 5% CO2 for 24 h, the growth medium was replaced with fresh medium containing 0.125, 0.25 and 0.5 μg/mL CYN and incubated for 24, 48, 72 and 96 h. In each experiment, a positive control (0.5 μM STS for 24 h) and a vehicle control (0.05% methanol) were included. After the exposure, cells were harvested and fixed as described above (3.5 DSB detection-H2AX foci analysis). Fixed cells were centrifuged at 1200 rpm for 10 min, washed twice with ice cold 1× PBS and stained with 0.5 mL propidium iodide/RNAse staining buffer for 15 min at room temperature according to the manufacturer’s recommendations. Flow cytometric analysis was carried out on FACSCalibur (BD Biosciences PharmingenTM, San Diego, CA, USA). Changes in the distribution of cells through the phases of the cell cycle were analyzed in the FL2 channel, where 10^4^ events were recorded for each sample. The percentage of cells in G0/G1, S, and G2/M phases of the cell cycle were determined from FL2-A histograms using ModFit LT™ (version 3.3, for Windows 7) Verity Software House, Topsham, ME, USA. Analysis was performed on single cells, by elimination of cell aggregates by gating FL2-W versus FL2-A. Independent experiments were repeated 3-times.

### 3.8. Statistical Analysis

Statistical significance between treated groups and the control was determined by One-way analysis of variance and Dunnett’s Multiple Comparison Test, using GraphPad Prism 5 (GraphPad Software, San Diego, CA, USA). For the γH2AX foci analysis the statistical significance between treated groups and the vehicle control was determined with a linear mixed-effects model. Calculations were done with the statistical program R [41] and its packages reshape [42], ggplot2 [43] and nlme [44].

## 4. Conclusions

Based on the results of the present study we can conclude that CYN is genotoxic for HepG2 cells and potentially presents a serious health risk for humans, as the toxin induced DNA double strand breaks at non-cytotoxic concentrations and reduced cell-proliferation of HepG2 cells by induction of cell cycle arrest in G0/G1 phase after 24 h of exposure and in S phase after prolonged exposure (72 and 96 h). The results of the present study show potential mechanisms of action of cyanotoxin CYN and present important findings especially when considering long term human exposure to the toxin. Although the concentrations used in the present study seem to be rather high they can be found in the environment. Environmental CYN concentrations were reported in the range between 9 and 18 µg/L [45], while in pond water samples CYN concentrations can reach up to 0.6 mg/L [46].

## References

[1] Moreira C., Azevedo J., Antunes A., Vasconcelos V. (2013). Cylindrospermopsin: Occurrence, methods of detection and toxicology. J. Appl. Microbiol..

[2] Ohtani I., Moore R.E., Runnegar M.T.C. (1992). Cylindrospermopsin: A potent hepatotoxin from the blue-green alga *Cylindrospermopsis raciborskii*. J. Am. Chem. Soc..

[3] Krohn A.J., Wahlbrink T., Prehn J.H.M. (1999). Mitochondrial depolarization is not required for neuronal apoptosis. J. Neurosci..

[4] Žegura B., Štraser A., Filipič M. (2011). Genotoxicity and potential carcinogenicity of cyanobacterial toxins—A review. Mutat. Res. Rev. Mutat. Res..

[5] Hawkins P.R., Runnegar M.T., Jackson A.R., Falconer I.R. (1985). Severe hepatotoxicity caused by the tropical cyanobacterium (blue-green alga) *Cylindrospermopsis raciborskii* (Woloszynska) Seenaya and Subba Raju isolated from a domestic water supply reservoir. Appl. Environ. Microbiol..

[6] Froscio S.M., Humpage A.R., Burcham P.C., Falconer I.R. (2003). Cylindrospermopsin-induced protein synthesis inhibition and its dissociation from acute toxicity in mouse hepatocytes. Environ. Toxicol..

[7] Bazin E., Mourot A., Humpage A.R., Fessard V. (2010). Genotoxicity of a freshwater cyanotoxin, cylindrospermopsin, in two human cell lines: Caco-2 and HepaRG. Environ. Mol. Mutagen..

[8] Humpage A., Fontaine F., Froscio S., Burcham P., Falconer I. (2005). Cylindrospermopsin genotoxicity and cytotoxicity: Role of cytochrome P-450 and oxidative stress. J. Toxicol. Environ. Health A.

[9] Humpage A.R., Fenech M., Thomas P., Falconer I.R. (2000). Micronucleus induction and chromosome loss in transformed human white cells indicate clastogenic and aneugenic action of the cyanobacterial toxin, cylindrospermopsin. Mutat. Res. Genet. Toxicol. Environ. Mutagen..

[10] Štraser A., Filipič M., Žegura B. (2011). Genotoxic effects of the cyanobacterial hepatotoxin cylindrospermopsin in the HepG2 cell line. Arch. Toxicol..

[11] Žegura B., Gajski G., Štraser A., Garaj-Vrhovac V. (2011). Cylindrospermopsin induced DNA damage and alteration in the expression of genes involved in the response to DNA damage, apoptosis and oxidative stress. Toxicon.

[12] Shaw G.R., Seawright A.A., Moore M.R., Lam P.K.S. (2000). Cylindrospermopsin, a cyanobacterial alkaloid: Evaluation of its toxicologic activity. Ther. Drug Monit..

[13] Shen X., Lam P.K.S., Shaw G.R., Wickramasinghe W. (2002). Genotoxicity investigation of a cyanobacterial toxin, cylindrospermopsin. Toxicon.

[14] Falconer I.R., Humpage A.R. (2001). Preliminary evidence for *in vivo* tumour initiation by oral administration of extracts of the blue-green alga *Cylindrospermopsis raciborskii* containing the toxin cylindrospermopsin. Environ. Toxicol..

[15] US Environmental Protection Agency (2010). Creating a Cyanotoxin Target List for the Unregulated Contaminant Monitoring Ruley.

[16] Bartek J., Lukas C., Lukas J. (2004). Checking on DNA damage in S phase. Nat. Rev. Mol. Cell Biol..

[17] Salaün P., le Breton M., Morales J., Bellé R., Boulben S., Mulner-Lorillon O., Cormier P. (2004). Signal transduction pathways that contribute to CDK1/cyclin B activation during the first mitotic division in sea urchin embryos. Exp. Cell Res..

[18] O’Farrell P.H. (2001). Triggering the all-or-nothing switch into mitosis. Trends Cell Biol..

[19] Štraser A., Filipič M., Žegura B. (2013). Cylindrospermopsin induced transcriptional responses in human hepatoma HepG2 cells. Toxicol. In Vitro.

[20] Štraser A., Filipič M., Gorenc I., Žegura B. (2013). The influence of cylindrospermopsin on oxidative DNA damage and apoptosis induction in HepG2 cells. Chemosphere.

[21] Fessard V., Bernard C. (2003). Cell alterations but no DNA strand breaks induced *in vitro* by cylindrospermopsin in CHO K1 cells. Environ. Toxicol..

[22] Lankoff A., Wojcik A., Lisowska H., Bialczyk J., Dziga D., Carmichael W.W. (2007). No induction of structural chromosomal aberrations in cylindrospermopsin-treated CHO-K1 cells without and with metabolic activation. Toxicon.

[23] Dasika G.K., Lin S.C., Zhao S., Sung P., Tomkinson A., Lee E.Y. (1999). DNA damage-induced cell cycle checkpoints and DNA strand break repair in development and tumorigenesis. Oncogene.

[24] Habraken Y., Piette J. (2006). NF-κB activation by double-strand breaks. Biochem. Pharmacol..

[25] Norbury C.J., Hickson I.D. (2001). Cellular Responses to DNA damage. Annu. Rev. Pharmacol. Toxicol..

[26] Rogakou E.P., Pilch D.R., Orr A.H., Ivanova V.S., Bonner W.M. (1998). DNA double-stranded breaks induce histone H2AX phosphorylation on serine 139. J. Biol. Chem..

[27] Sedelnikova O.A., Rogakou E.P., Panyutin I.G., Bonner W.M. (2002). Quantitative detection of (125)IdU-induced DNA double-strand breaks with gamma-H2AX antibody. Radiat. Res..

[28] Rogakou E.P., Boon C., Redon C., Bonner W.M. (1999). Megabase chromatin domains involved in DNA double-strand breaks *in vivo*. J. Cell Biol..

[29] Michael D., Oren M. (2002). The p53 and Mdm2 families in cancer. Curr. Opin. Genet. Dev..

[30] Bruno S., Darzynkiewicz Z. (1992). Cell cycle dependent expression and stability of the nuclear protein detected by Ki-67 antibody in HL-60 cells. Cell Prolif..

[31] Levine A.J. (1997). p53, the cellular gatekeeper for growth and division. Cell.

[32] Ko L.J., Prives C. (1996). p53: Puzzle and paradigm. Genes Dev..

[33] Bain P., Shaw G., Patel B. (2007). Induction of p53-regulated gene expression in human cell lines exposed to the cyanobacterial toxin cylindrospermopsin. J. Toxicol. Environ. Health A.

[34] Shimizu N., Itoh N., Utiyama H., Wahl G.M. (1998). Selective entrapment of extrachromosomally amplified DNA by nuclear budding and micronucleation during S phase. J. Cell Biol..

[35] Yankiwski V., Marciniak R.A., Guarente L., Neff N.F. (2000). Nuclear structure in normal and bloom syndrome cells. Proc. Natl. Acad. Sci. USA.

[36] Fenech M. (2000). The *in vitro* micronucleus technique. Mutat. Res. Genet. Toxicol. Environ. Mutagen..

[37] Mosmann T. (1983). Rapid colorimetric assay for cellular growth and survival: Application to proliferation and cytotoxicity assays. J. Immunol. Methods.

[38] Zegura B., Zajc I., Lah T.T., Filipic M. (2008). Patterns of microcystin-LR induced alteration of the expression of genes involved in response to DNA damage and apoptosis. Toxicon.

[39] FCSExtract Utility for Flow Cytometry Standard (FCS) Data. Earl F Glynn Stowers Institute for Medical Research. http://research.stowers-institute.org/efg/ScientificSoftware/Utility/FCSExtract/index.htm.

[40] Hreljac I., Zajc I., Lah T., Filipič M. (2008). Effects of model organophosphorous pesticides on DNA damage and proliferation of HepG2 cells. Environ. Mol. Mutagen..

[41] R Development Core Team (2012). R: A Language and Environment for Statistical Computing.

[42] Wickham H. (2007). Reshaping data with the reshape package. J. Stat. Softw..

[43] Wickham H. (2009). ggplot2: Elegant Graphics for Data Analysis.

[44] Pinheiro J., Bates D., DebRoy S., Sarkar D. (2012). R Development Core Team (2012) nlme: Linear and Nonlinear Mixed Effects Models.

[45] Klitzke S., Fastner J. (2012). Cylindrospermopsin degradation in sediments—The role of temperature, redox conditions, and dissolved organic carbon. Water Res..

[46] Saker M.L., Eaglesham G.K. (1999). The accumulation of cylindrospermopsin from the cyanobacterium *Cylindrospermopsis raciborskii* in tissues of the Redclaw crayfish *Cherax quadricarinatus*. Toxicon.

